# Sub-minute acquisition with deep learning-based image filter in the diagnosis of colorectal cancers using total-body ^18^F-FDG PET/CT

**DOI:** 10.1186/s13550-023-01015-z

**Published:** 2023-07-10

**Authors:** Entao Liu, Zejian Lyu, Yuelong Yang, Yang Lv, Yumo Zhao, Xiaochun Zhang, Taotao Sun, Lei Jiang, Zaiyi Liu

**Affiliations:** 1 WeiLun PET Center, Department of Nuclear Medicine, Guangdong Provincial People’s Hospital (Guangdong Academy of Medical Sciences), Southern Medical University, Guangzhou, China; 2 Department of Gastrointestinal Surgery, Department of General Surgery, Guangdong Provincial People’s Hospital (Guangdong Academy of Medical Sciences), Southern Medical University, Guangzhou, China; 3 Department of Radiology, Guangdong Provincial People’s Hospital (Guangdong Academy of Medical Sciences), Southern Medical University, Room 201, 2/F, WeiLun Building of Guangdong Provincial People’s Hospital, 106 Zhongshan Er Road, Guangzhou, 510080 Guangdong China; 4 Guangdong Provincial Key Laboratory of Artificial Intelligence in Medical Image Analysis and Application, Guangzhou, China; 5 United Imaging Healthcare, Shanghai, China

**Keywords:** Total-body PET/CT, Colorectal cancer, Fast acquisition, Diagnostic performance

## Abstract

**Background:**

This study aimed to retrospectively evaluate the feasibility of total-body ^18^F-FDG PET/CT ultrafast acquisition combined with a deep learning (DL) image filter in the diagnosis of colorectal cancers (CRCs).

**Methods:**

The clinical and preoperative imaging data of patients with CRCs were collected. All patients underwent a 300-s list-mode total-body ^18^F-FDG PET/CT scan. The dataset was divided into groups with acquisition durations of 10, 20, 30, 60, and 120 s. PET images were reconstructed using ordered subset expectation maximisation, and post-processing filters, including a Gaussian smoothing filter with 3 mm full width at half maximum (3 mm FWHM) and a DL image filter. The effects of the Gaussian and DL image filters on image quality, detection rate, and uptake value of primary and liver metastases of CRCs at different acquisition durations were compared using a 5-point Likert scale and semi-quantitative analysis, with the 300-s image with a Gaussian filter as the standard.

**Results:**

All 34 recruited patients with CRCs had single colorectal lesions, and the diagnosis was verified pathologically. Of the total patients, 11 had liver metastases, and 113 liver metastases were detected. The 10-s dataset could not be evaluated due to high noise, regardless of whether it was filtered by Gaussian or DL image filters. The signal-to-noise ratio (SNR) of the liver and mediastinal blood pool in the images acquired for 10, 20, 30, and 60 s with a Gaussian filter was lower than that of the 300-s images (*P* < 0.01). The DL filter significantly improved the SNR and visual image quality score compared to the Gaussian filter (*P* < 0.01). There was no statistical difference in the SNR of the liver and mediastinal blood pool, SUVmax and TBR of CRCs and liver metastases, and the number of detectable liver metastases between the 20- and 30-s DL image filter and 300-s images with the Gaussian filter (*P* > 0.05).

**Conclusions:**

The DL filter can significantly improve the image quality of total-body ^18^F-FDG PET/CT ultrafast acquisition. Deep learning-based image filtering methods can significantly reduce the noise of ultrafast acquisition, making them suitable for clinical diagnosis possible.

**Supplementary Information:**

The online version contains supplementary material available at 10.1186/s13550-023-01015-z.

## Background

Recently, new PET/CT scanners with long-axial field of view (LAFOV) and silicon photomultiplier (SiPM) detection systems have been introduced, such as the axial FOV of 194-cm PET/CT (uEXPLORER, United Imaging Healthcare Co), 106-cm PET/CT (Biograph Vision Quadra, Siemens Healthineers), and 64-cm PET/CT (PennPET Explorer) scanners [1–4]. These LAFOV PET/CT scanners are characterised by increased sensitivity owing to their ability to collect more photons during scanning, allowing reduced tracer injection doses and shortened acquisition time.

Shorter acquisition durations are desirable for the comfort of patients, particularly those who are distressed, claustrophobic, have shortness of breath, are children, or require less dosage of anaesthetic; shorter acquisition durations is also cost-effective. One of the challenges in routine PET/CT is to deal with patients with dyspnoea in a recumbent position or severe pain following bone metastases. In such cases, maintaining diagnostic performance whilst achieving fast PET acquisitions would be especially beneficial.

However, shortening the acquisition time may result in increased noise, lower signal-to-noise ratio (SNR), and potentially unnecessary image artefacts [5]. These factors may affect the quality and accuracy of the images, potentially compromising the diagnosis and treatment planning for the patient, especially for the detection of liver metastases or interference of physiological accumulation of ^18^F-FDG by the adjacent colon in colorectal cancer (CRC). Benign FDG uptake in the colon on PET/CT indicates physiological uptake, inflammation (such as inflammatory bowel disease), and benign lesions (such as benign polyps). This can affect the detection and diagnosis of CRCs, especially when the difference between tumour and colon benign uptake is not significant or when there is noise interference.

The advent of deep learning-based image filters has the potential to decrease noise and enhance image quality in short-term image acquisition, such as HYPER DLR launched by United Imaging Healthcare and licenced by the US Food and Drug Administration (FDA) 510(k) clearance. HYPER DLR, a deep learning-based algorithm for PET image filters, can effectively remove noise from images captured under low count rate conditions, significantly improving image quality. This technology can boost image SNRs by 42% and increase imaging speed [6].

Xing et al. attempted to use HYPER DLR to reduce image noise and achieved good results, but did not evaluate the image quality with an acquisition time of less than 1 min [6]. Some researchers have explored ultrafast PET acquisition and attempted to evaluate PET image quality within 1 min [7–10]. However, image noise caused by short-term acquisition using a Gaussian filter has affected the diagnosis, with standard detectors covering an axial field of view [11]. Although these studies on the ultrafast acquisition of PET/CT have revealed that acquisition speed has significantly improved, studies on the quality of images using a Gaussian filter still need to be completed. Further to this, previous studies have included a variety of diseases, but there is a lack of research on CRC, specifically in patients with liver metastasis. The effect of liver noise on the ability to detect metastatic tumours remains unknown.

Therefore, considering the 300-s OSEM reconstruction image with a 3 mm Gaussian smoothing filter as a standard image, we retrospectively compared the effects of the Gaussian and DL image filters on image quality, detection rate, and uptake rate of primary and metastatic CRCs in total-body PET/CT imaging at different acquisition durations (10, 20, 30, 60, and 120 s).

## Methods

### Study design and population

The local Institutional Review Board approved this retrospective study (No. KY2023-020-01) and waived the requirement for informed consent. From April 2022 to December 2022, 34 consecutive patients with CRCs were enrolled in this study.

### Inclusion criteria

Patients were included in this study based on the following inclusion criteria: (i) no previous history of malignancies; (ii) the diagnosis was confirmed by histopathology; and (iii) only received symptomatic treatment and had no history of chemotherapy, radiotherapy, or surgical resection before the PET/CT scan.

### Exclusion criteria

Patients were excluded for the following reasons: (i) incomplete image datasets and (ii) lack of a final histological diagnosis.

### Patient preparation and PET/CT protocol

All patients fasted for more than 4 h before ^18^F-FDG injection according to the EANM procedure guidelines for tumour imaging (version 2.0) [12]. All patients underwent implantation of 22 G indwelling intravenous catheters (Jierui Medical Product), followed by ^18^F-FDG manual administration with 2.96 MBq/kg. The patients were instructed to lie on the bed as calmly as possible. Imaging was started 60 ± 5 min after ^18^F-FDG injection. Image acquisition was performed using a total-body PET/CT scanner (uEXPLORER, United Imaging Healthcare, Shanghai, China) with an axial FOV of 194 cm. Additional file 1: Table 1 lists the parameters of the PET component of the PET/CT scanner. Low-dose CT was performed before PET for attenuation correction and anatomical localisation with a dose-modulation technique. Subsequently, total-body PET imaging was performed using a 3D list-mode with 300-s acquisition for one-bed position.

PET images were initially reconstructed with OSEM using data from the full 300-s acquisition. Images were post-processed using a 3 mm isotropic Gaussian smoothing filter. The necessary correction methods were applied, such as attenuation and scatter correction. Subsequently, the PET images were reconstructed using various acquisition times (10, 20, 30, 60, and 120 s) to simulate fast scans with both Gaussian and DL image filters. The parameters used in the OSEM reconstruction process included time of flight (TOF) and point spread function (PSF) modelling, three iterations, 20 subsets, 600 cm field of view, a matrix size of 192 × 192, a pixel size of 3.125 × 3.125 × 2.886 mm^3^, and a Gaussian post-filter of 3 mm FWHM. For the DL image filter process, the Gaussian post-filter was replaced, whereas all other reconstruction parameters were the same as described above.

### Imaging analysis

All images were transferred to a workstation (uWS-MI:R002, United Imaging Healthcare) and reviewed in standard planes. Taking the 300-s image with a Gaussian filter image as a standard image, Gaussian and DL filtering images with five image datasets (10, 20, 30, 60, and 120 s) were included for comparison.

For qualitative analysis, the image quality of various time-point PET/CT datasets was evaluated visually by two nuclear physicians (Xiaochun Zhang and Taotao Sun) with over a decade of experience in PET/CT diagnosis. According to the widely used 5-point Likert scale, the image quality was scored, and the criteria were as follows: (i) very poor image quality and excessive noise (score 1); (ii) poor image quality and increased noise (score 2); (iii) fair image quality, similar to the regular image of daily practice (score 3); (iv) good image quality, superior to the regular image of daily practice (score 4); and (v) excellent image quality with minimal noise (score 5) [13–17]. The two readers were blinded to the evaluation of the various time-point PET/CT dataset images and scored.

To eliminate intra-observer variability in the quantitative analysis, PET/CT images were quantitatively evaluated by a single nuclear physician with over a decade of experience in PET/CT diagnosis. Semi-automatic 3D delineation of the FDG-avid lesions was performed to cover the entire tumour. 3D isocontour volume of interest (VOI) based on 41% of the maximum standardised uptake value (SUVmax) thresholds was used and recommended by EANM guidelines [12]. The tumour VOIs were obtained with the 300-s OSEM reconstruction with Gaussian (3 mm FWHM) filter and subsequently replicated and applied to other PET acquisition datasets of images. Mean standardised uptake value (SUVmean), maximum standardised uptake value (SUVmax), and peak standardised uptake value (SUVpeak) within a 1-cm^3^ spherical volume were automatically generated.

According to the recommendations of PERCIST, hepatic ^18^F-FDG activity was assessed using a fixed 3-cm-diameter spherical VOI on the right lobe of the liver [18, 19]. Additionally, ^18^F-FDG activity in the mediastinal blood pool was evaluated using a cylindrical VOI with a diameter of 1 cm and a long axis of 2 cm (parallel to the descending aorta) at the centre of the descending thoracic aorta. The SUVmean and standard deviation (SD) of the liver and mediastinal blood pool were recorded. The liver and mediastinal blood pool SNRs were calculated by dividing SUVmean by SD. The calculation formula used was as follows:

$${\text{Liver}}\;{\text{SNR}} = \frac{{{\text{SUVmean}}\;{\text{of}}\;{\text{Liver}} }}{{{\text{SD}}}}$$

$${\text{Mediastinal}}\;{\text{blood}}\;{\text{pool}}\;{\text{SNR}} = \frac{{{\text{SUVmean}}\;{\text{of}}\;{\text{Mediastinal}}\;{\text{blood}}\;{\text{pool}}}}{{{\text{SD}}}}$$

The tumour-to-background ratio (TBR) was calculated by dividing the SUVmax of the tumour by the SUVmean of the liver. The calculation formula used was as follows:

$${\text{TBR}} = \frac{{{\text{SUVmax}}\;{\text{of}}\;{\text{tumour}} }}{{{\text{SUVmean}}\;{\text{of}}\;{\text{Liver}} }}$$

To evaluate the detectability of the primary lesion of CRCs, the SUVmax of the adjacent proximal and distal bowel of the tumour was measured, and the tumour-to-adjacent bowel ratio (TAR) was calculated. The calculation formula used was as follows:

$${\text{TAR}} = \frac{{{\text{SUVmax}}\;{\text{of}}\;{\text{tumour}}}}{{{\text{SUVmax}}\;{\text{of}}\;{\text{the}}\;{\text{surrounding}}\;{\text{bowel}}\;{\text{of}}\;{\text{tumour}} }}$$

For patients with multiple liver metastases, the number of liver metastases was found on the 300-s image with a Gaussian filter image as a standard image reference, and the analysis focused on the largest and smallest lesions on the standard reconstruction. The liver metastases TBR and detection rate of liver metastases were calculated. The calculation formula used was as follows:

$${\text{Liver}}\;{\text{metastases}}\;{\text{TBR}} = \frac{{{\text{SUVmax}}\;{\text{of}}\;{\text{Liver}}\;{\text{metastases}}}}{{{\text{SUVmean}}\;{\text{of}}\;{\text{Liver}}}}$$

### Statistical analysis

Continuous variables were presented as mean ± SD, and categorical variables were presented as frequencies and percentages. The weighted Kappa statistic was applied to evaluate inter-observer agreement for different acquisition durations of the PET/CT datasets image scores. The value of the agreement was categorised as follows: no agreement (κ < 0), slight agreement (0 ≤ κ < 0.2), fair agreement (0.21 ≤ κ < 0.4), moderate agreement (0.41 ≤ κ < 0.6), substantial agreement (0.61 ≤ κ < 0.8), and excellent agreement (0.81 ≤ κ ≤ 1). The Friedman’s test with post hoc comparisons using Bonferroni correction was used to compare differences in SNR, TBR, and tumour SUVs among various time-point PET/CT dataset images. Statistical analyses were performed using SPSS (v.26.0), GraphPad Prism (v.9.0.0), and MedCalc (v.19.0.7). A two-tailed probability value of < 0.05 was considered statistically significant.

## Results

### Population

From April 2022 to December 2022, 34 consecutive patients with a single CRC lesion, histopathologically confirmed, were enrolled in this study. Of the total patients, 11 had liver metastases, and 113 liver metastases were detected. All 11 patients underwent triple-phase abdominal contrast-enhanced CT (CECT), and the size of the liver metastases was measured using enhanced CT. Patient characteristics are shown in Table 1. The distribution of the primary tumour sites in CRCs is shown in Additional file 2: Fig. 1.

**Table 1:** The characteristics of the patients

Characteristics	Value
Number of patients	34
Age (years)	67 (32–93)
Gender, n (%)	
Male	20
Female	14
Weight (kg)	56.9 ± 12.0 (35.0–85.0)
Height (cm)	161.4 ± 8.5 (143.0–181.0)
BMI (kg/m^2^)	21.7 ± 3.4 (15.6–30.1)
Fasting blood glucose level (mg/dl)	105.4 ± 18.8 (73.8–160.2)
^18^F-FDG injection dose (MBq)	168.4 ± 35.5 (103.6–251.6)
CEA (ng/ml)	74.5 ± 185.3 (0.92–1033.0)
Delay time (min)	60 ± 4.3 (51–72)
Location (number of patients)	
Rectal cancer	1
Colon cancer	33
Ascending	11
Transverse	5
Descending	10
Sigmoid	7
TNM	
I-II	6
III	11
IV	17
Primary tumour	
SUVmax	21.6 ± 13.9 (7.3–82.7)
TBR	8.8 ± 4.9 (2.9–26.7)
Liver metastases	
SUVmax	8.4 ± 5.2 (3.2–22.7)
TBR	3.7 ± 2.2 (1.5–8.4)
Size	
Largest lesion	4.6 ± 2.7 (1.0–8.5)
Minimal lesion	0.9 ± 0.4 (0.6–2.1)

### Qualitative assessment of image quality and inter-observer agreement

**Fig. 1:**
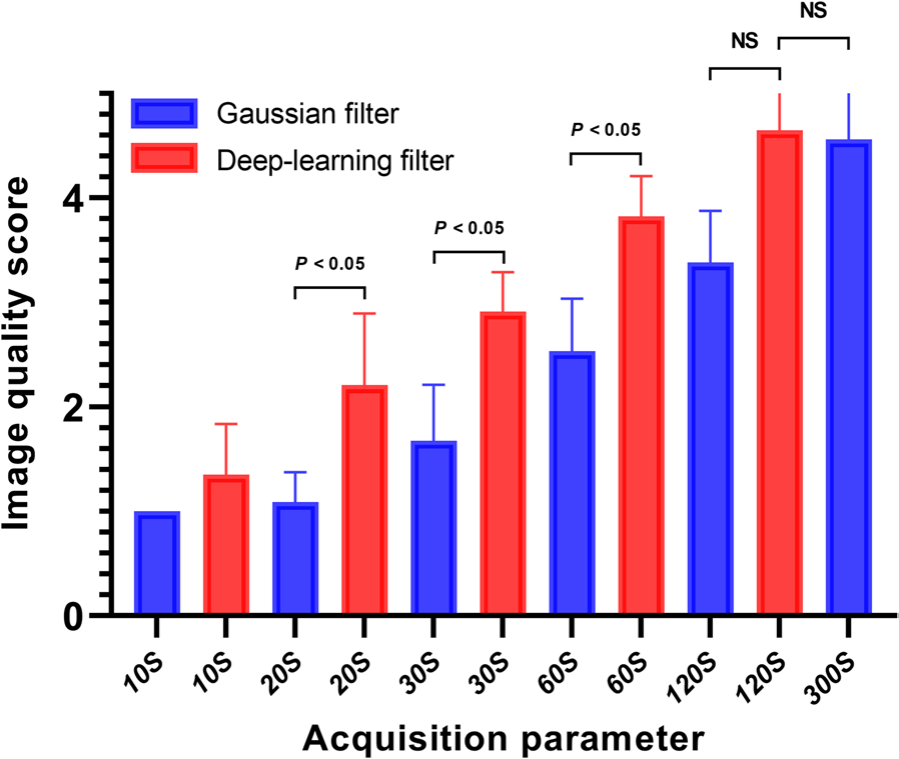
The image quality score of PET image reconstructed using Gaussian and DL image filter for different acquisition durations. DL image filter significantly improves visual image quality scores of 20-, 30-, and 60-s acquisition time images compared to Gaussian filter (*P* < 0.01). There is no statistical difference in the image quality score between the 120-s Gaussian or DL image filter image and the 300-s Gaussian filter image (*P* > 0.05)

Based on a 5-point Likert scale, the weighted kappa coefficient for the inter-observer agreement of the image quality evaluation was 0.822 (95% confidence interval, 0.655–0.989). The 10-, 20-, and 30-s acquisition durations images with Gaussian filter were scored 1 or 2, and none were scored 3. DL image filter significantly improved the visual image quality scores of 20-, 30-, and 60-s acquisition time images compared to Gaussian filter (*P* < 0.01) (Additional file 3: Fig. 2 and Additional file 4: Fig. 3). The scores of PET image using Gaussian and DL image filter for different acquisition durations are shown in Fig. 1.

### Quantitative assessment of image quality

**Fig. 2:**
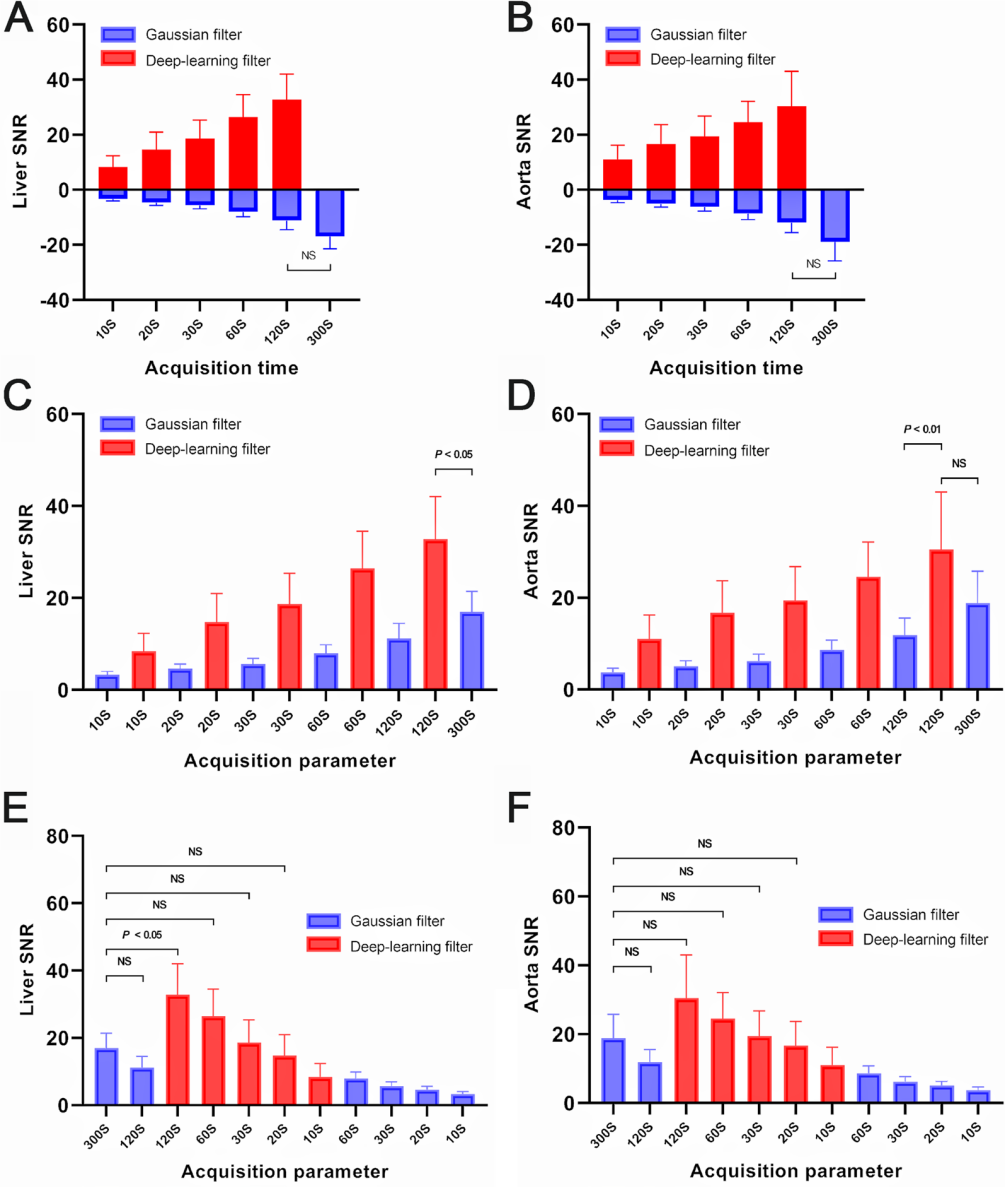
The SNR of different acquisition durations images with Gaussian and DL image filter. The SNR of the liver (**A**) and mediastinal blood pool (**B**, measured at descending aorta) of the DL image filter image with different acquisition durations is higher than that of the Gaussian filter image (*P* < 0.01). **C** and **D** show that the SNR improves steadily as the acquisition time increases, for both Gaussian and DL image filter images. There is no statistical difference in the SNR of the liver (**E**) and mediastinal blood pool (**F**) between the 20- and 30-s DL image filter image and the 300-s Gaussian filter image (*P* > 0.05)

The 10-s dataset could not be evaluated due to high noise, regardless of whether it was filtered by Gaussian or DL image filters. The SNR of the liver and mediastinal blood pool in the images acquired for 10, 20, 30, and 60 s with a Gaussian filter was lower than that of the 300-s images (*P* < 0.01) (Additional file 5: Fig. 4). The DL filter significantly improved the SNR and visual image quality score compared to the Gaussian filter (*P* < 0.01). There was no statistical difference in the SNR of the liver (*P* = 0.176 and *P* = 0.635) and mediastinal blood pool (*P* = 0.257 and *P* = 0.942) between the 20- and 30-s DL image filter and 300-s images with the Gaussian filter. The SNR of different acquisition duration images with Gaussian and DL image filters is shown in Fig. 2, Table 2, and Table 3.

**Table 2:** The SNR, SUVmax, and TBR of different acquisition durations images with Gaussian and deep learning filter

Parameter	Gaussian filter						Deep learning filter				
	10S	20S	30S	60S	120S	300S	10S	20S	30S	60S	120S
SNR											
Liver	3.3 ± 0.7	4.6 ± 1.0	5.6 ± 1.2	8 ± 1.9	11.1 ± 3.3	17.0 ± 4.4	8.4 ± 4.0	14.7 ± 6.2	18.7 ± 6.7	26.4 ± 8.1	32.8 ± 9.2
Aorta	3.7 ± 0.9	5.1 ± 1.2	6.2 ± 1.5	8.6 ± 2.2	11.9 ± 3.7	18.8 ± 6.9	11.0 ± 5.2	16.7 ± 6.9	19.4 ± 7.3	24.6 ± 7.5	30.5 ± 12.5
SUVmax											
Lesion of CRCs	26.1 ± 14.8	24.0 ± 14.0	23.4 ± 13.9	22.8 ± 14.5	22.3 ± 14.8	21.6 ± 13.9	21.7 ± 13.4	20.4 ± 13.1	19.9 ± 12.9	19.5 ± 13.3	19.1 ± 13.4
Liver metastases	10.3 ± 6.0	9.6 ± 5.9	9.4 ± 5.8	8.8 ± 5.3	8.5 ± 5.1	8.4 ± 5.2	8.3 ± 5.2	7.8 ± 5.2	7.6 ± 5.0	7.3 ± 4.9	7.2 ± 4.9
TBR											
Lesion of CRCs	10.5 ± 5.3	9.7 ± 5.0	9.4 ± 4.9	9.2 ± 5.1	9.0 ± 5.2	8.8 ± 4.9	8.7 ± 4.5	8.2 ± 4.6	8.1 ± 4.5	7.9 ± 4.6	7.8 ± 4.7
Liver metastases	4.5 ± 2.7	4.2 ± 2.6	3.9 ± 2.3	3.8 ± 2.3	3.7 ± 2.2	3.7 ± 2.2	3.6 ± 2.3	3.9 ± 2.2	3.3 ± 2.2	3.2 ± 2.1	3.2 ± 2.1
TAR	6.5 ± 4.1	6.9 ± 4.4	6.9 ± 4.2	7.1 ± 4.1	7.1 ± 4.2	7.5 ± 4.4	7.4 ± 4.9	7.5 ± 5.0	7.4 ± 5.0	7.2 ± 4.7	7.1 ± 4.6

**Table 3:** Quantitative comparison of 20- and 30-s deep learning filter and 300-s Gaussian filter images

	Gaussian filter	Deep learning filter		*P* value
	300S	20S	30S	
SNR				
Liver	17.0 ± 4.4	14.7 ± 6.2	18.7 ± 6.7	*P* > 0.05
Aorta	18.8 ± 6.9	16.7 ± 6.9	19.4 ± 7.3	*P* > 0.05
SUVmax				
Lesion of CRCs	21.6 ± 13.9	20.4 ± 13.1	19.9 ± 12.9	*P* > 0.05
Liver metastases	8.4 ± 5.2	7.8 ± 5.2	7.6 ± 5.0	*P* > 0.05
TBR				
Lesion of CRCs	8.8 ± 4.9	8.2 ± 4.6	8.1 ± 4.5	*P* > 0.05
Liver metastases	3.7 ± 2.2	3.9 ± 2.2	3.3 ± 2.2	
TAR	7.5 ± 4.4	7.5 ± 5.0	7.4 ± 5.0	*P* > 0.05

### Quantitative assessment of colorectal cancer and liver metastases

**Fig. 3:**
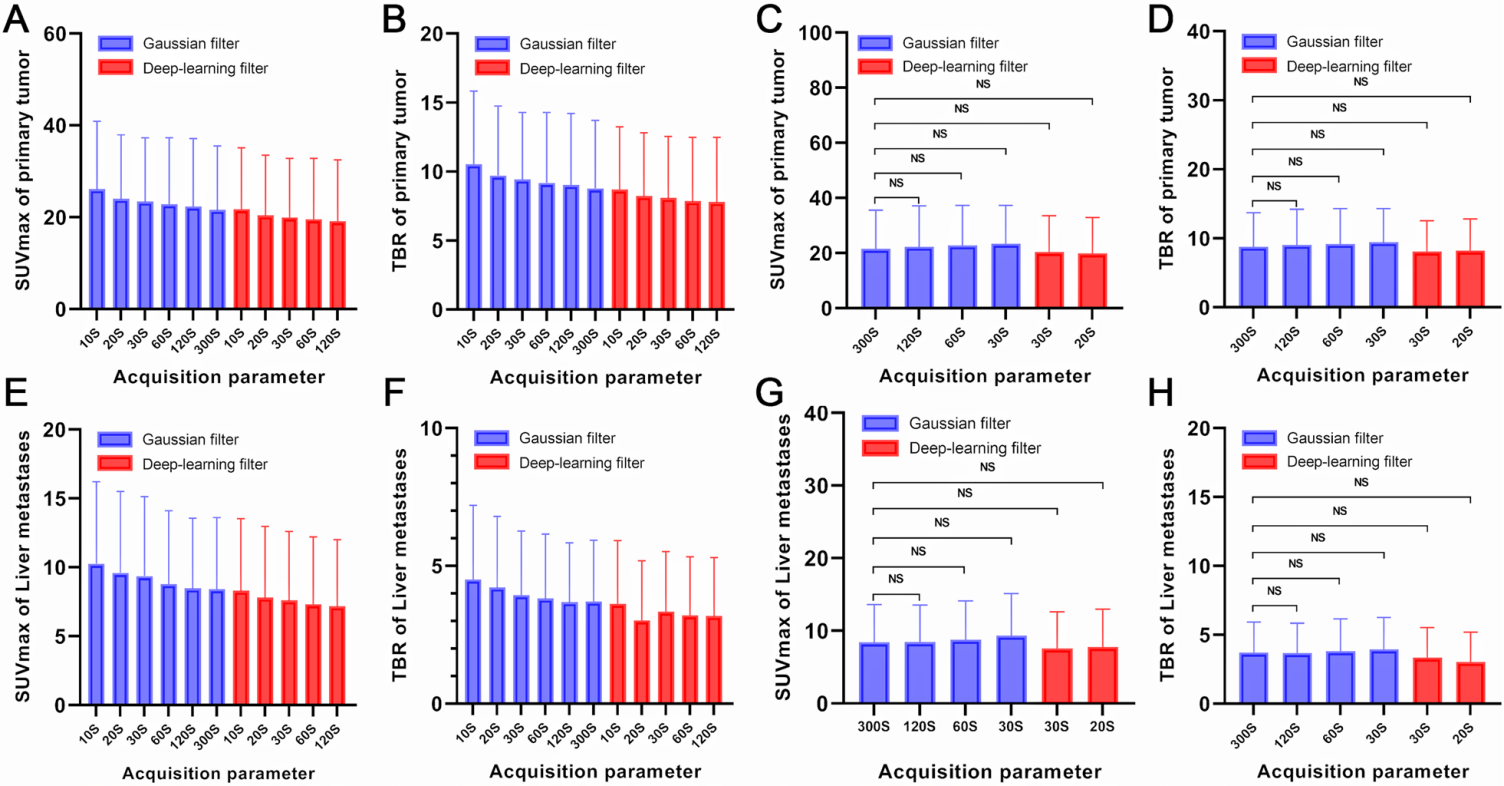
The SUVmax and TBR of CRCs and liver metastases of different acquisition durations images with Gaussian and DL image filter. The SUVmax (**A**) and TBR (**B**) of CRCs showed gradual increases with decreasing acquisition times. There are no statistical difference in SUVmax (**C**) and TBR (**D**) of CRCs between the 20- and 30-s DL image filter image and the 300-s Gaussian filter image (*P* > 0.05). **E**, **F**, **G**, and **H** show the same findings in liver metastases

The SUVmax and TBR of CRCs and liver metastases gradual increased with decreasing acquisition times. There were no statistical differences in the SUVmax (*P* = 0.961 and *P* = 0.071) and TBR (*P* = 0.189 and *P* = 0.081) of CRCs and liver metastases between the 20- and 30-s DL image filter and 300-s images with the Gaussian filter. The SUVmax and TBR of different acquisition durations of images with Gaussian and DL image filters are shown in Fig. 3, Tables 2, and Table 3. The SUVmax and TBR of the 60- and 120-s images with DL image filter were lower than those of the 300-s images with Gaussian filter (*P* < 0.01) (Additional file 6: Fig. 5). A comparison of all quantitative data is presented in Additional file 7: Table 2.

To evaluate the detectability of primary lesions of CRCs, we introduced TAR, which is the ratio of the primary tumour to tumour–adjacent bowel SUVmax. There was no statistical difference in the TAR (*P* = 0.324,* P* = 0.306,* P* = 0.125, and *P* = 0.073) between the Gaussian and DL image filter images with different acquisition durations (20, 30, 60, and 120 s) and the 300-s images with Gaussian filter image.

**Fig. 4:**
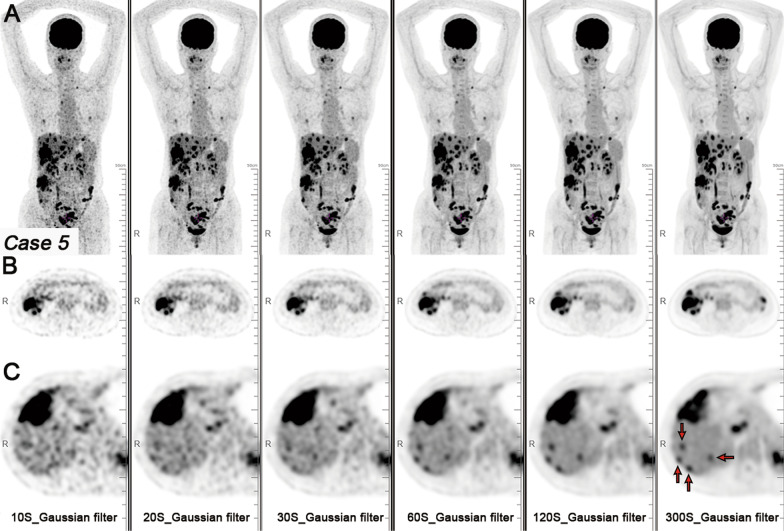
A patient with CRC and liver metastases. Maximum intensity projection (MIP) PET images (**A**), axial PET images of colon cancer (**B**), and axial PET images of liver metastases (**C**) with Gaussian filter with different acquisition durations (10, 20, 30, 60, 120, and 300 s). The 10-, 20-, and 30-s acquisition durations images with Gaussian filter exhibited noticeable noise, making it difficult to observe small liver metastases (red arrow). As the acquisition duration was extended, the liver metastases were clearly displayed

**Fig. 5:**
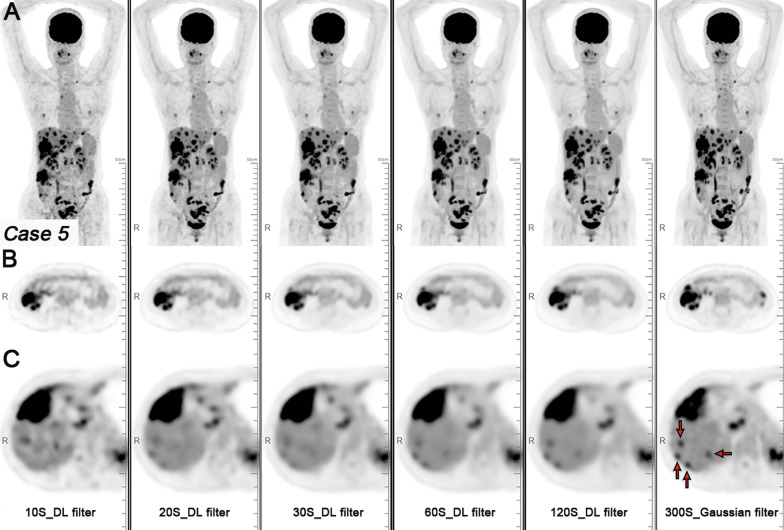
The same patient. MIP PET images (**A**), axial PET images of colon cancer (**B**), and axial PET images of liver metastases (**C**) with DL image filter with different acquisition durations (10, 20, 30, 60, and 120 s). With DL image filter, the noise of the image is significantly reduced and the image quality is significantly improved. With the 300-s Gaussian filter image as a standard image reference, sub-centimetre liver metastases (red arrow) become blurred on DL image filter image

With the 300-s images with Gaussian filter image as a standard image reference, a total of 113 liver metastases were detected. The 10- and 20-s acquisition durations images with Gaussian filter exhibited noticeable noise, making it difficult to observe small liver metastases (Fig. 4). Sub-centimetre liver metastases were not conducive to display on the DL image filter image (Fig. 5). Compared with the 300-s images with Gaussian filter images, the detection rate of liver metastases on 60 and 120-s images with DL image filter decreased. In terms of the number of detectable liver metastases, the 10- and 20-s acquisition durations images with Gaussian filter were significantly lower than the 300-s images with Gaussian filter images (*P* < 0.05). There was no statistical difference in the number of detectable liver metastases between the 20- and 30-s images with DL filter and the 300-s images with Gaussian filter image (*P* = 0.077 and *P* = 0.123).

## Discussion

Our current study shows that DL image filter can significantly improve the image quality and SNR for low count data. Without affecting the quantitative evaluation of CRCs or liver metastases, the acquisition time of total-body PET/CT can be reduced to 20 s using DL image filter. For the visual qualitative evaluation of image quality, DL image filter significantly improved the image quality score of 20-, 30-, and 60-s acquisition time images compared with Gaussian filter (*P* < 0.01). Visually, there was no difference in image quality between DL image filter images and 300-s images with Gaussian filter images (Additional file 8: Fig. 6). For the SNR of the liver and mediastinal blood pool, compared with Gaussian filter, DL image filter can increase the SNR of 20-s, 30-s, and 60-s datasets images by three times (Fig. 3A and 3B). Because of the 10-s datasets, whether Gaussian filter or DL image filter was too poor in SNR, it was not considered. Compared with the 300-s images with Gaussian filter images, the SNR of the 20- and 30-s images with DL image filter was similar.

When the acquisition time for PET/CT imaging is reduced, the image noise level tends to increase significantly. The SUVmax of CRC and liver metastatic lesions also tends to increase gradually. This observation applies to both images processed with a Gaussian filter and DL image filter. However, it is noted that the increase in SUVmax is relatively lower when using the DL image filter compared to the Gaussian filter. The detection of sub-centimetre liver metastases of CRCs is still a problem that puzzles PET/CT daily work. It is worth noting that DL image filter makes it difficult to detect sub-centimetre liver metastases from CRCs.

In this study, we compared the data from similar studies [9]. The results are presented in Table 4. Compared with the current study, the SNR of the liver and mediastinal blood pool in Zhang et al.’s study is higher than that of our image data, whether it is 30- or 300-s image with a Gaussian filter. We speculate that this is caused by different FDG doses (3.7 vs. 2.96 MBq/kg).

**Table 4:** Quantitative comparison based on 300-s Gaussian filter image and comparison with relevant study

		Gaussian filter		*P* value	Gaussian filter		Deep learning filter	*P* value
		300S	30S		300S	30S	20S	
The current study	SNR							
	Liver	17.0 ± 4.4	5.6 ± 1.2	*P* < 0.01	17.0 ± 4.4	18.7 ± 6.7	14.7 ± 6.2	*P* > 0.05
	Aorta	18.8 ± 6.9	6.2 ± 1.5	*P* < 0.01	18.8 ± 6.9	19.4 ± 7.3	16.7 ± 6.9	*P* > 0.05
	SUVmax	21.6 ± 13.9	23.4 ± 13.9	0.063	21.6 ± 13.9	19.9 ± 12.9	20.4 ± 13.1	*P* > 0.05
	TBR	8.8 ± 4.9	9.4 ± 4.9	0.418	8.8 ± 4.9	8.1 ± 4.5	8.2 ± 4.6	*P* > 0.05
Zhang et al	SNR							
	Liver	19.71 ± 5.58	9.03 ± 2.51	*P* < 0.01				
	Aorta	16.60 ± 6.48	10.43 ± 3.1	*P* < 0.01				
	SUVmax	13.94 ± 11.83	15.48 ± 14.19	0.003				
	TBR	4.43 ± 3.61	4.54 ± 3.80	0.411				

Meanwhile, we also found that the SNR of the 20- and 30-s DL image filter images was higher than that of Zhang et al.’s 30-s Gaussian filter image [9]. Additionally, the SNR of 30-s DL image filter image was similar to that of Zhang et al.’s 300 s with a Gaussian filter image [9]. It has been demonstrated that the SNR of DL image filter images is better than that of Gaussian filter by a 30-s ultrafast acquisition by total-body PET/CT. And the 30-s DL image filter images was equivalent to the 300-s reconstruction images. The SUV and TBR of CRCs in this study were significantly higher than those in Zhang et al.’s study, presumably because of the different types of tumours included.

The increase in image noise due to fast acquisition may affect the detection and diagnosis of CRCs, especially when the difference between tumour and benign colon uptake is not significant or noise interference. Our study also evaluated the detectability of CRC lesions, compared the difference between CRC lesions and adjacent bowel benign FDG uptake, and used the SUVmax of lesions and adjacent bowel uptake. The shortened acquisition time led to deviations in SUVmax caused by noise.

Significantly, compared with the 300-s image with a Gaussian filter images, we have shortened the acquisition time and are more friendly to patients who cannot tolerate the conventional PET/CT acquisition time. In the routine management of CRCs, ultrafast acquisition may be a practical substitute for 300-s PET/CT acquisition.

Additionally, our study found that the SUVmax of the Gaussian filter image datasets acquired for more than 60 s was consistent with the results of the datasets acquired for 5 min (Fig. 4). Our results are consistent with Tan et al.’s research on assessing CRCs with data collected at 1–5 different minute time points using the same equipment [20]. However, these results differ from those of Sher et al., who found that the SUVmax of images collected at 1.5 min differed from those collected at 5 min [21]. This may be due to differences in equipment (long-axial FOV PET detectors and standard PET detectors) and the composition of the study cases, which are not focused on CRCs (mainly lymphoma and lung cancer).

### Limitations of our study

The limitations of this study include its small sample size and retrospective design. One limitation of this study is that only one patient had a BMI greater than 30, defined as obesity. It is well known that obesity affects SNR and reduces the SNR of fast-acquisition PET images. This study also did not qualitatively and quantitatively evaluate the nearby lymph nodes of colorectal cancer, which will be the focus of the next step.

## Conclusions

The DL filter can significantly improve the image quality of total-body ^18^F-FDG PET/CT ultrafast acquisition. Deep learning-based image filtering methods can significantly reduce the noise of ultrafast acquisition, making them suitable for clinical diagnosis possible.

## Supplementary Information

**Additional file 1: Table 1.** Parameters of the PET component of PET/CT scanner

**Additional file 2: Figure 1.** Distribution of the primary tumour sites of CRCs. Each circle represents a single patient. Of the 34 CRC cases, 11 were located in the ascending colon, five in the transverse colon, 10 in the descending colon, seven in the sigmoid colon, and one in the rectum

**Additional file 3: Figure 2.** A patient with CRC and liver metastases. Maximum intensity projection (MIP) PET images (**A**), anteroposterior MIP PET images (**B**), and oblique MIP PET images (**C**) with Gaussian filter with different acquisition durations (10, 20, 30, 60, 120, and 300 second). **B** and **C**, partial enlargement of MIP PET images for displaying the liver metastases and colon cancer (white arrow). As the acquisition duration was extended, the noise in the image gradually decreased

**Additional file 4: Figure 3.** The same patient was on the 10-, 20-, 30-, 60-, and 120-second DL filter image. MIP PET images (**A**), anteroposterior MIP PET images (**B**), and oblique MIP PET images (**C**) with DL image filter for displaying the liver metastases and colon cancer (white arrow). DL image filter significantly improves the visual image quality of 20-, 30-, and 60-second acquisition time images compared to a 300-second Gaussian filter

**Additional file 5: Figure 4.** The SNR of different acquisition durations images with Gaussian and DL image filters. The SNR of the liver (**A**) and mediastinal blood pool (**B**) of 10-, 20-, 30-, and 60-second acquisition durations images reconstructed with the same reconstruction parameters is lower than that of 300-second Gaussian filter images (*P* ＜ 0.01)

**Additional file 6: Figure 5.** The SUVmax and TBR of different acquisition durations images with Gaussian and DL image filters. The SUVmax (**A**) and TBR (**B**) of the 60- and 120-second DL image filter images were lower than those of the 300-second Gaussian filter image (*P* ＜ 0.01)

**Additional file 7: Table 2.** Comparison of SNR, SUVmax, and TBR of images with different acquisition durations reconstructed by Gaussian filter and deep learning filter

**Additional file 8: Figure 6.** With the 300-second Gaussian filter image as a standard image reference, the comparison of the image quality between the 10-, 20-, and 30-second Gaussian and DL image filter in some cases. **A**–**H** The partial enlargement of MIP PET images to display the colon cancer (white arrow)

## Data Availability

All data in the current study are available from the corresponding author on reasonable request.

## Abbreviations

CECT: Contrast-enhanced CT
CRC: Colorectal cancer
FOV: Field of view
LAFOV: Long-axial field of view
OSEM: Ordered subset expectation maximisation
PSF: Point spread function
SNR: Signal-to-noise ratio
SiPM: Silicon photomultiplier
SD: Standard deviation
SUVmax: Standardised uptake value
SUVmean: Mean standardised uptake value
SUVpeak: Peak standardised uptake value
TAR: Tumour-to-adjacent bowel ratio
TBR: Tumour-to-background ratio
TOF: Time of flight
