# Supplementary material for: Sub-minute acquisition with deep learning-based image filter in the diagnosis of colorectal cancers using total-body 18F-FDG PET/CT
Source: EJNMMI Res. 2023 Jul 10;13:66. doi: 10.1186/s13550-023-01015-z (PMC10333161; doi:10.1186/s13550-023-01015-z)
Supplement: Supplementary file 1 — Additional file 1: Table 1. Parameters of the PET component of PET/CT scanner [file 13550_2023_1015_MOESM1_ESM.docx]

| Supplementary Table 1. Parameters of the PET component of PET/CT scanner | |
| --- | --- |
| Characteristics | Description |
| Scintillator | Lutetium–yttrium oxyorthosilicate |
| Photodetectors | Silicon photomultiplier |
| Number of detector rings | 672 |
| Total number of crystals | 564,480 |
| Crystal Size (mm^3^) | 2.72 × 2.76 × 18 |
| Axial Field of View (cm) | 194 |
| Detector Ring Diameter (cm) | 78.6 |
| Transverse Field of View (cm) | 70.0 |
| Coincidence time Resolution (ps) | 430 |
| Energy Resolution | 11.7% |
| Maximum axial (polar) angle | 57° |
| Spatial Resolution (mm) | 2.9 |
| Sensibility (kcps/MBq) | 176 |
| Matrix (maximal value) | 1024 × 1024 |
