# Supplementary material for: Sub-minute acquisition with deep learning-based image filter in the diagnosis of colorectal cancers using total-body 18F-FDG PET/CT
Source: EJNMMI Res. 2023 Jul 10;13:66. doi: 10.1186/s13550-023-01015-z (PMC10333161; doi:10.1186/s13550-023-01015-z)
Supplement: Supplementary file 7 — Additional file 7: Table 2. Comparison of SNR, SUVmax, and TBR of images with different acquisition durations reconstructed by Gaussian filter and deep learning filter [file 13550_2023_1015_MOESM7_ESM.docx]

Supplementary Table 2. Comparison of SNR, SUVmax, and TBR of images with different acquisition durations reconstructed by Gaussian filter and Deep-learning filter

| Parameter | Duration | Gaussian filter | Deep-learning filter | *P* value |
| --- | --- | --- | --- | --- |
| SNR |  |  |  |  |
| Liver | 10S | 3.3 ± 0.7 | 8.4 ± 4.0 | *P* < 0.01 |
|  | 20S | 4.6 ± 1.0 | 14.7 ± 6.2 | *P* < 0.01 |
|  | 30S | 5.6 ± 1.2 | 18.7 ± 6.7 | *P* < 0.01 |
|  | 60S | 8 ± 1.9 | 26.4 ± 8.1 | *P* < 0.01 |
|  | 120S | 11.1 ± 3.3 | 32.8 ± 9.2 | *P* < 0.01 |
|  | 300S | 17.0 ± 4.4 |  |  |
| Aorta | 10S | 3.7 ± 0.9 | 11.0 ± 5.2 | *P* < 0.01 |
|  | 20S | 5.1 ± 1.2 | 16.7 ± 6.9 | *P* < 0.01 |
|  | 30S | 6.2 ± 1.5 | 19.4 ± 7.3 | *P* < 0.01 |
|  | 60S | 8.6 ± 2.2 | 24.6 ± 7.5 | *P* < 0.01 |
|  | 120S | 11.9 ± 3.7 | 30.5 ± 12.5 | *P* < 0.01 |
|  | 300S | 18.8 ± 6.9 |  |  |
| SUVmax |  |  |  |  |
| Lesion of CRCs | 10S | 26.1 ± 14.8 | 21.7 ± 13.4 | *P* < 0.01 |
|  | 20S | 24.0 ± 14.0 | 20.4 ± 13.1 | *P* < 0.01 |
|  | 30S | 23.4 ± 13.9 | 19.9 ± 12.9 | *P* < 0.01 |
|  | 60S | 22.8 ± 14.5 | 19.5 ± 13.3 | *P* < 0.01 |
|  | 120S | 22.3 ± 14.8 | 19.1 ± 13.4 | *P* < 0.01 |
|  | 300S | 21.6 ± 13.9 | |  |
| Liver metastases | 10S | 10.3 ± 6.0 | 8.3 ± 5.2 | *P* < 0.01 |
|  | 20S | 9.6 ± 5.9 | 7.8 ± 5.2 | *P* < 0.01 |
|  | 30S | 9.4 ± 5.8 | 7.6 ± 5.0 | *P* < 0.01 |
|  | 60S | 8.8 ± 5.3 | 7.3 ± 4.9 | *P* < 0.01 |
|  | 120S | 8.5 ± 5.1 | 7.2 ± 4.9 | *P* < 0.01 |
|  | 300S | 8.4 ± 5.2 |  |  |
| TBR |  |  |  |  |
| Lesion of CRCs | 10S | 10.5 ± 5.3 | 8.7 ± 4.5 | *P* < 0.01 |
|  | 20S | 9.7 ± 5.0 | 8.2 ± 4.6 | *P* < 0.01 |
|  | 30S | 9.4 ± 4.9 | 8.1 ± 4.5 | *P* < 0.01 |
|  | 60S | 9.2 ± 5.1 | 7.9 ± 4.6 | *P* < 0.01 |
|  | 120S | 9.0 ± 5.2 | 7.8 ± 4.7 | *P* < 0.01 |
|  | 300S | 8.8 ± 4.9 |  |  |
| Liver metastases | 10S | 4.5 ± 2.7 | 3.6 ± 2.3 | *P* < 0.01 |
|  | 20S | 4.2 ± 2.6 | 3.9 ± 2.2 | *P* < 0.01 |
|  | 30S | 3.9 ± 2.3 | 3.3 ± 2.2 | *P* < 0.01 |
|  | 60S | 3.8 ± 2.3 | 3.2 ± 2.1 | *P* < 0.01 |
|  | 120S | 3.7 ± 2.2 | 3.2 ± 2.1 | *P* < 0.01 |
|  | 300S | 3.7 ± 2.2 |  |  |
| TAR* |  |  |  |  |
|  | 10S | 6.5 ± 4.1 | 7.4 ± 4.9 | *P* > 0.05 |
|  | 20S | 6.9 ± 4.4 | 7.5 ± 5.0 | *P* > 0.05 |
|  | 30S | 6.9 ± 4.2 | 7.4 ± 5.0 | *P* > 0.05 |
|  | 60S | 7.1 ± 4.1 | 7.2 ± 4.7 | *P* > 0.05 |
|  | 120S | 7.1 ± 4.2 | 7.1 ± 4.6 | *P* > 0.05 |
|  | 300S | 7.5 ± 4.4 |  |  |

* TAR, the CRCs tumor-to-adjacent bowel ratio
